# The association between ambient air pollution and birth defects in four cities in Hunan province, China, from 2014 to 2016

**DOI:** 10.1097/MD.0000000000014253

**Published:** 2019-01-25

**Authors:** Lili Xiong, Zenghui Xu, Hua Wang, Zhiyu Liu, Donghua Xie, Aihua Wang, Fanjuan Kong

**Affiliations:** a Hunan Province Maternal and Children Care Hospital, Changsha, China; b Changsha Environment Protection College, Changsha, China.

**Keywords:** air pollution, birth defects, Hunan province, NO_2_, PM_10_, SO_2_

## Abstract

This study was performed to assess whether air pollution was positively associated with birth defects and if a specific pregnancy stage played a role. This was a population-based case-control study comprising 153,822 perinatal births in four cities located in Hunan province, China, during the period 2014 to 2016. Exposure to SO_2_, NO_2_, and PM_10_ in each pregnant woman in the first 3 months before pregnancy, and in the first and third trimester was assessed. The risk of birth defects related to SO_2_ in the first 3 months before pregnancy was between 1.191 and 1.566. In the first trimester stage the risk was between 1.104 and 1.348. The risk of birth defects related to NO_2_ before pregnancy was 1.285 (95%CI: 1.180–1.399), in the first trimester stage the risk was between 1.280 (95%CI: 1.197–1.368) and 1.380 (95%CI: 1.293–1.473). In the third month before delivery the risk was 1.484 (95%CI: 1.366–1.613). The risk of birth defects related to PM_10_ in the first month of pregnancy was 1.098 (95%CI: 1.057–1.140), and in the third month before delivery the risk was 1.296 (95%CI: 1.222–1.375). SO_2_ had a greater effect on the prophase of pregnancy, while NO_2_ and PM_10_ had an effect in the late third trimester.

## 1 Introduction

Birth defects as well as congenital anomalies, congenital disorders, and congenital malformations can be defined as structural or functional anomalies (e.g., metabolic disorders) that occur during intrauterine life and can be identified prenatally, at birth or later in life. Birth defects can contribute to long-term disability, which may have a significant impact on individuals, families, health-care systems, and societies. Birth defects accounted for 510,400 deaths worldwide in 2010, a total of 1% of all deaths, and ranked 23rd among all causes of death.^[1]^ Worldwide, an estimated 303,000 newborns die within 4 weeks of birth due to birth defects in each year.^[2]^ Birth defects are present in approximately 2.03% in the United States based a population study.^[3]^ In China, the estimated prevalence is approximately 4% to 6%.^[4]^ The prevalence rates of birth defects in Hunan province ranked third in China in 2011, fourth in 2012, and fifth in 2013.

Birth defects can occur during any stage of pregnancy. Most birth defects occur in the first 3 months of pregnancy, when the baby's organs are formed. This is a very important stage of development. However, some birth defects occur later in pregnancy, as the tissues and organs continue to grow and develop in the last 6 months of pregnancy. Maternal exposure to air pollution may increase the risk of birth defects. Many studies investigating the effect of maternal exposure to ambient air pollution on birth defects have been conducted worldwide^[5–9]^. China has been experiencing exceptionally high levels of air pollution in recent years due to the booming economy. Therefore, an increasing number of studies have examined the effects of air pollution on birth defects in China.^[10–14]^ The study in Xi’an, China, used a generalized additive model to investigate the relationship between birth defects and ambient air pollutants, showed nitrogen dioxide (NO_2_) increased risk of neural tube defects, congenital heart disease, congenital polydactyly, cleft palate, digestive system abnormalities and gastroschisis, and PM_10_ was associated with congenital heart disease and cleft lip with or without cleft palate.^[10]^ The hospital-based case-control study in Fuzhou, China, showed some positive associations between maternal exposure to ambient particles with an aerodynamic diameter of 10 mm or less (PM_10_) during the first 2 months of pregnancy and fetal cardiovascular malformations.^[13]^ The retrospective cohort study in Anqing city, Eastern China, suggested that exposure to ambient sulfur dioxide (SO_2_) during pregnancy may increase the risk of birth defects.^[12]^ The study in Lanzhou, China, investigated a cohort of 8969 singleton live births by using inverse distance weighting way for exposure assessment found positive associations for congenital malformations of cardiac septa with PM_10_ exposures in the second trimester and the entire pregnancy, and SO_2_ exposures in the entire pregnancy.^[11]^ In a word, there were differences in the studies in the selection of subgroups of birth defects, exposure window, exposure assessment method and adjustments for confounding factors, and the representativeness problem that each study conducted in different areas.

In light of the inconclusive evidence on the occurrence of birth defects in the different stages of pregnancy, as well as the lack of studies in provincial areas in China with a high prevalence of birth defect, we conducted a study in Hunan province, China in order to obtain data on these issues. Therefore, the objective of this study was to investigate whether maternal exposure to SO_2_, NO_2_, and PM_10_ was associated with an elevated risk of birth defects during three pregnancy periods (prophase of pregnancy, pregnancy and late pregnancy) in Hunan province, China. This study is the first to investigate the relationship between birth defects and ambient air pollution at a provincial level in China.

## 2 Materials and methods

### 2.1 Study design

We used a population-based case-control study design to examine the association between ambient pollution and birth defects in Hunan province, China. Hunan province is located in central China, covers 21.18 km^2^, and has a population of 71.47 million people, with 14 cities and 123 counties. We selected the following four cities, Changsha, Changde, Yongzhou, and Huaihua, which are located in central, north, south, and west of Hunan province respectively, and are selected as the air monitoring stations for nation. All birth defect monitoring hospitals in the four cities were selected. In total, 18 birth defect monitoring hospitals were included. The study period was from October 1, 2013 to September 30, 2016.

### 2.2 Study subjects and data resources

The study subjects were perinatal births including live births, stillbirths, and deaths within 7 days, and excluded twins or multiple births and family genetic history in the 18 birth defect monitoring hospitals in the four cities in Hunan province from October 1, 2013 to September 30, 2016. All the data were sourced from the Hunan province women and children health direct report management system, which included the birth defect registration card, the seasonal report on the number of perinatal births, and childbirth record. All the data was exported under the supervision of the health administration. Therefore, the study and the consent procedure were approved by the Medical Ethics Committee of Hunan province Maternal and Children Care Hospital.

The birth defect registration card included information on maternal conditions, child conditions, birth defect diagnosis, early pregnancy, and family history. Maternal conditions included present address, ethnicity, age, cultural level, family income, number of pregnancies, and total previous live births. Child conditions included date of birth, sex, gestational age, number of children, weight, and outcome. Birth defects included 24 types were classified according to the International Classification of Diseases Clinical Modification Codes, tenth edition, (ICD-10) as congenital malformations, deformations, and chromosomal abnormalities (Codes Q00–Q99). Early pregnancy conditions included illness, drug administration and exposure to other harmful factors. Family history included abnormal reproductive history and a detailed family history. The childbirth record included maternal name, age, address, gestational week, gravidity, parity, delivery mode, and newborn conditions. The health monitoring data report was mainly the health administration data as the number of perinatal births. All data were eventually submitted to the relevant national departments after quality control by provincial relevant departments.

### 2.3 Cases and controls

Cases were those with reported birth defects during the study period, and controls were a 25% random sample of live births born during the study period, without birth defects and sufficient information for the indicators. Pregnancies with missing childbirth record information or implausible gestational age information (<20 weeks or >44 weeks) were excluded.

### 2.4 Exposure assessment

The daily concentrations of ambient air pollutants (NO_2_, SO_2_, and PM_10_) from October 1, 2013 to September 30, 2016 were obtained from the Environment Protection Bureau of the corresponding city, which in all covered from 167 monitoring stations. Daily, monthly, and seasonal average levels of the three pollutants were computed based on these measurements. The concentrations of SO_2_, NO_2_, and PM_10_ were assigned to each maternal residence based on the nearest monitor to the residential address reported at the time of the first routine physical examination. On average, there were three monitoring stations in each county. Three monitoring stations were located predominantly in the central districts of each county. We estimated the date of conception by adding 14 days to the date of last menses.

### 2.5 Covariates

Potential covariates from the birth defects records, which included gender of infant (male, female), maternal age (<20 years, 20–34 years, ≥35 years), total previous live births (0, 1, and ≥2), number of pregnancies (1, 2, and ≥3), and season of conception (spring, summer, fall, and winter), were entered for the final adjustments based on the previous studies and the data available.

### 2.6 Statistical analysis

We adopted binomial logistic regression to examine the association between SO_2_, NO_2_, and PM_10_ and the risk of birth defects by calculating the odds ratio (OR) and 95% confidence intervals (CI). The *χ*^2^ test was conducted to examine the distributions of selected characteristics in the cases and controls. Percentile values were used to describe the pollutant concentration distribution. Microsoft Excel 2010 was used to enter data and SPSS 17.0 software was used for statistical analysis.

## 3 Results

### 3.1 Description of birth defects

Table 1 shows the birth defect rate in the four cities in Hunan province from October 1, 2013 to September 30, 2016. There were 153,822 perinatal births observed in 18 birth defect monitoring hospitals. Among them, 5021 cases had birth defects and the birth defect rate was 3.26%. Totally 38,179 controls were sourced from 25% of the total perinatal births in this study. The incidence of birth defects in the four regions was different and statistically significant (*χ*^2^ = 368.01, *P* < .01). Table 2 shows the frequent birth defect types. Congenital heart defect was the most common (*N* = 1498) and represented 29.83% of all birth defects.

**Table 1:**
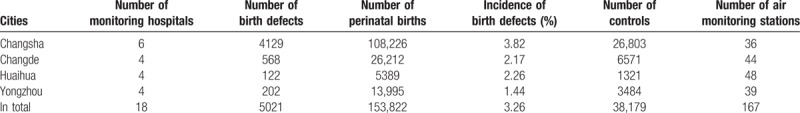
The incidence of birth defects in four cities in Hunan province, China from October 1, 2013 to September 30, 2016.

**Table 2:**
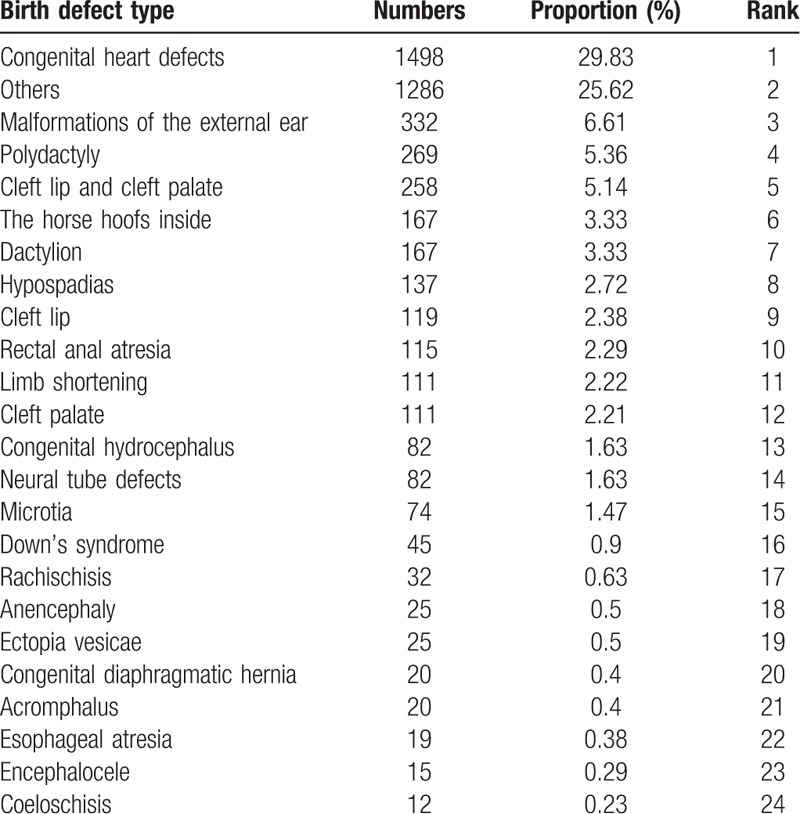
Proportions of the 24 birth defect types in Hunan province, China from October 1, 2013 to September 30, 2016.

### 3.2 Characteristics of the control and case subjects

The characteristics of the control group and the case group are presented in Table 3. Infants with or without birth defects showed differences between the areas, maternal age, infant gender, season of conception, total previous live births, and number of pregnancies (*P* < .001). These variables were considered as the confounding factors.

**Table 3:**
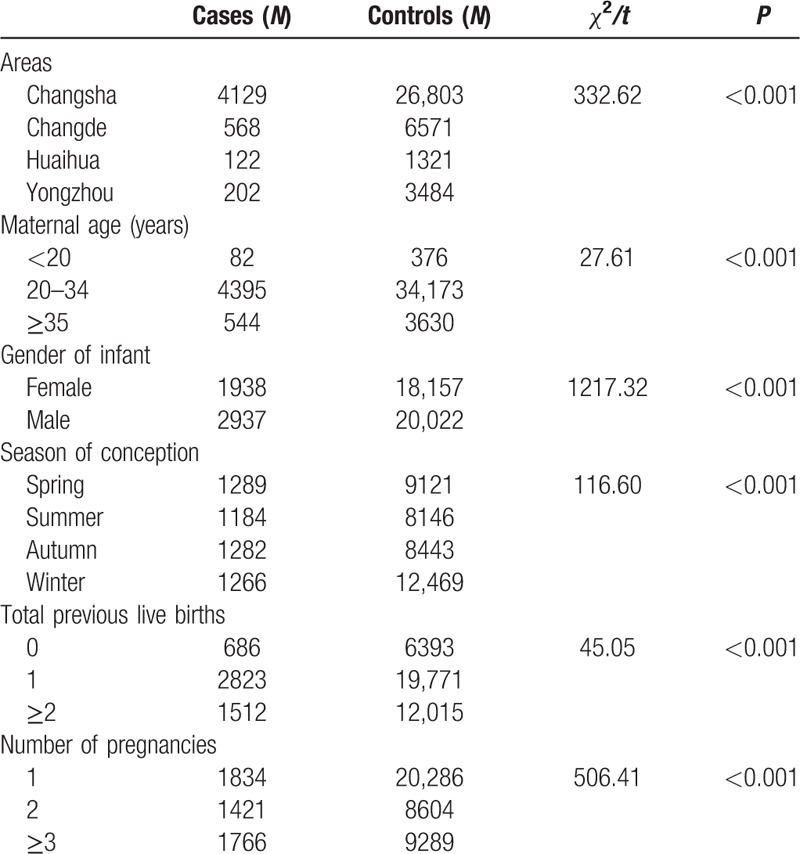
Characteristics of the study subjects in the four cities in Hunan province, China from October 1, 2013 to September 30, 2016.

### 3.3 Air pollutant concentrations

Descriptive statistics of the concentrations of air pollutants during the study period are shown in Table 4. The level of SO_2_ was highest in Yongzhou city, in 2014, and in winter (*P* < .001). The level of NO_2_ was highest in Changsha area, in 2014, and in winter (*P* < .001). The level of PM_10_ was highest in Changde area, in 2015, and in winter (*P* < .001).

**Table 4:**
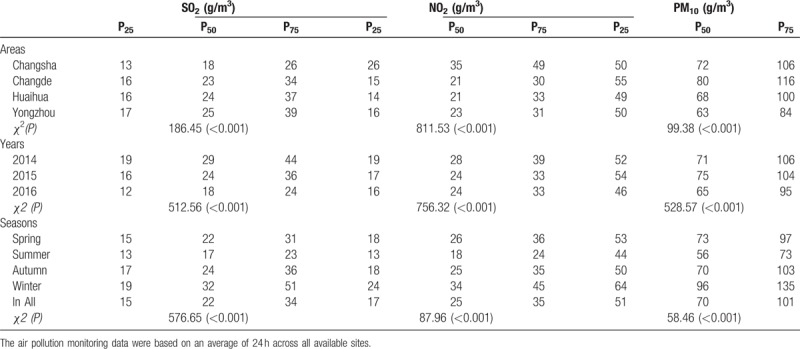
Air pollution levels in four cities in Hunan province, China from October 1, 2013 to September 30, 2016.

### 3.4 SO_2_ and the risk of birth defects

Table 5 shows the concentration of SO_2_ in the prophase of pregnancy, and in the first and third trimester, and summarizes the results of logistic regression analysis with/without adjusting for areas, year, season of conception, maternal age, gender of infant, total previous live births and number of pregnancies in the three pregnancy stages. There was difference between controls and cases for concentration of SO_2_ in the study period (*P* < .001). The concentration of SO_2_ was highest in the prophase of pregnancy. In the simple factor regression model without adjusting any variables, SO_2_ was a risk factor for birth defects in the first 3 months of each stage. In the multiple factor regression model, the risk of birth defects related to SO_2_ in the first to third month before pregnancy was 1.191 (95%CI: 1.107–2.202) to 1.566 (95%CI: 1.107–2.202), in the first to third month of pregnancy was 1.270 (95%CI: 1.202–1.342), 1.348 (95%CI: 1.264–1.438), and 1.104 (95%CI: 1.039–1.173), and in the second month before delivery was 1.130 (95%CI: 1.068–1.196).

**Table 5:**
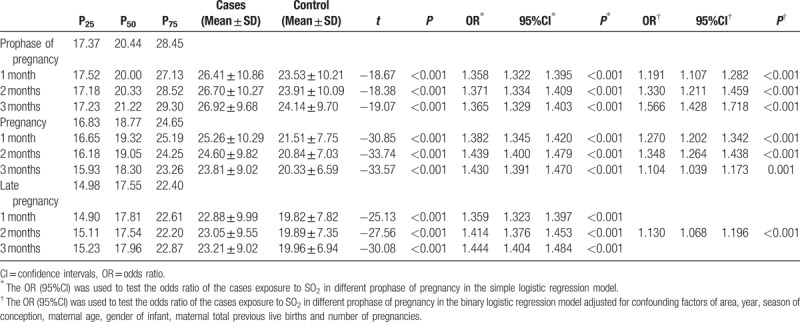
Effects of exposure to SO_2_ pollution on the risk of birth defects in different gestations.

### 3.5 NO_2_ and the risk of birth defects

Table 6 shows the concentration of NO_2_ in the prophase of pregnancy, and in the first and third trimester, and summarizes the results of logistic regression analysis with/without adjusting for covariates in the three stages. There was difference between controls and cases for concentration of NO_2_ in the study period (*P* < .001). The concentration of NO_2_ was highest in the pregnancy stage. In the simple factor regression model without adjusting any variables, NO_2_ was a risk factor for birth defects in the first 3 months of each stage. In the multiple factor regression model, the risk of birth defects related to NO_2_ in the second month before pregnancy was 1.285 (95%CI: 1.180–1.399), in the second and third month of the first trimester was 1.280 (95%CI: 1.197–1.368) and 1.380 (95%CI: 1.293–1.473), and in the third month of the third trimester was 1.484 (95%CI: 1.366–1.613).

**Table 6:**
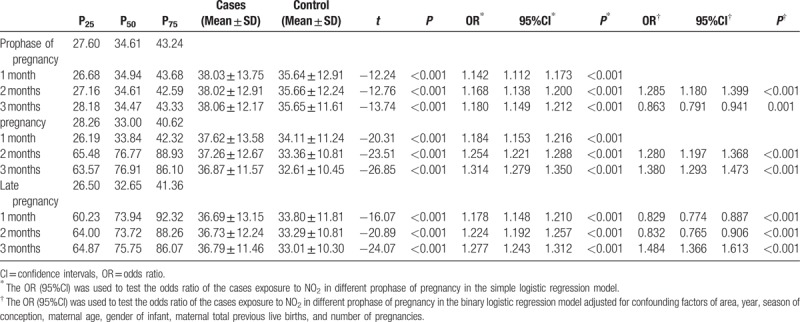
Effects of exposure to NO_2_ pollution on the risk of birth defects in different gestations.

### 3.6 PM_10_ and the risk of birth defects

Table 7 shows the concentration of PM_10_ in the prophase of pregnancy, and in the first and third trimester, and summarizes the results of logistic regression analysis with/without adjusting for covariates. There was difference between controls and cases for concentration of PM_10_ in the study period (*P* < .001). The concentration of PM_10_ was highest in the prophase of pregnancy. In the simple factor regression model without adjusting any variables, PM_10_ was a risk factor for birth defects in the first 3 months of each stage. In the multiple factor regression model, the risk of birth defects related to PM_10_ in the first month of the first trimester was 1.098 (95%CI: 1.057–1.140), and in the third month of the third trimester was 1.296 (95%CI: 1.222–1.375).

**Table 7:**
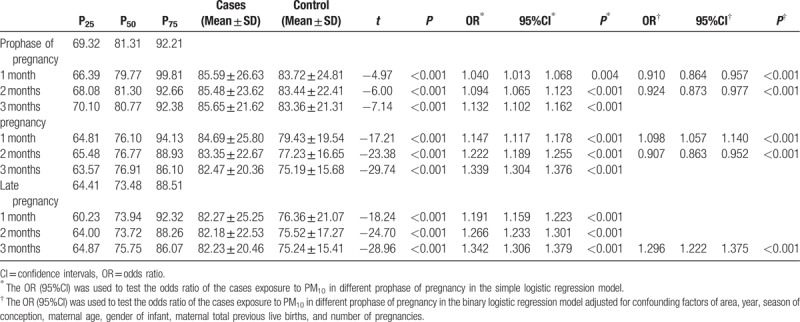
Effects of exposure to PM_10_ pollution on the risk of birth defects in different gestations.

## 4 Discussion

Over the past few decades, birth defects were the most common cause of infant mortality. The etiology of birth defects is thought to be multifactorial, with environmental exposure to air pollution suspected to play a role.^[15]^ Approximately 6%–8% of birth defects were associated with exposure to air pollution.^[16]^ As no studies have been carried out at the provincial level in China, and based on the inconclusive evidence on the effect of air pollution on birth defects in the stages of pregnancy, we conducted this study based on the four cities as the selected air monitoring stations for nation to determine the association between air pollution and birth defects.

In this population-based case-control study, we found a positive association between SO_2_ exposure and the prophase of pregnancy, pregnancy, and the second month before delivery following the adjustment of confounding factors. SO_2_ emissions are associated with coal combustion in power plants and industrial facilities.^[17]^ In the four selected cities, Huaihua and Yongzhou had higher SO_2_ concentrations as most energy enterprises are located in south and west Hunan province. The SO_2_ concentration was higher during autumn and winter as more energy is needed in cold seasons. Some studies on the effects of SO_2_ on birth defects have been conducted. For example, Martine Vrijheid conducted a systematic review and meta-analysis and concluded that SO_2_ exposure was related to an increased risk of coarctation of the aorta (OR = 1.07, 95%CI: 1.01–1.13) and tetralogy of fallot (OR = 1.03, 95%CI: 1.01–1.05).^[18]^ Dolk et al observed a significant association between SO_2_ and tetralogy of fallot.^[19]^ Giloba et al observed a significant association between SO_2_ and ventricular septal defects.^[20]^ Gianicolo et al found that exposure to the 90th percentile of SO_2_ increased the risk of congenital heart defects and ventricular septal defects.^[21]^ However, Esther Kai-Chieh Chen et al did not find that SO_2_ concentrations were significantly associated with birth defects.^[22]^ The difference maybe the methods used in the studies, such as case and control definition, exposure assessment, could affect the result.

This study showed that NO_2_ exposure during the second month of the prophase of pregnancy, the second and third month of pregnancy, and the third month before delivery was associated with birth defects. The NO_2_ concentration was higher in Changsha city and during the cold season as Changsha is a provincial capital city and has more vehicles and more energy is used during the cold season. Some studies have shown the effects of NO_2_ on certain types of birth defects. Esther Kai-Chieh Chen et al conducted a systematic review and meta-analysis and concluded that NO_2_ concentration was significantly associated with coarctation of the aorta (OR = 1.20, 95%CI: 1.02–1.41).^[22]^ Schembari et al concluded that NO_2_ was associated with coarctation of the aorta and digestive system defects.^[23]^ Dadvand et al showed an association between NO_2_ and congenital heart diseases, ventricular septal defects, cardiac septa malformations, and tetralogy of fallot.^[24]^ The different result also maybe the different methods used in the studies.

This study showed that PM_10_ exposure during the second month of pregnancy, and the third month before delivery was associated with birth defects. The PM_10_ concentration was higher in Changde city and during the winter. PM_10_ exposure level was an average of 101 μg/m^3^, similar to a recent report by Zhang et al, in which the mean PM_10_ level was 101.7 μg/m^3^.^[25]^ The China National Ambient Air Quality Surveillance Network showed that annual levels of PM_10_ in one-third of cities in China exceeded 100 μg/m^3^.^[26]^ A significant association between PM_10_ and patent ductus arteriosus was observed in a study by Strickland et al^[6]^ Dolk et al observed a significant association between PM_10_ and omphalocele.^[19]^ Kim et al showed that congenital anomalies were influenced by exposure to PM_10_.^[27]^ Gianicolo et al found that exposure to SO_2_ increased the risk of atrial septal defect.^[21]^

To date, the effects of air pollution in the pathogenesis of birth defects is unclear.^[16]^ Air pollutants may participate in the formation of skeletal muscle during fetal development through mechanisms such as hemodynamics, oxidative stress, and cytotoxicity. SO_2_ and its derivatives absorbed into the blood had adverse effects on germ cells and the development of embryos, and impaired the function of germ cells and their microstructure.^[28]^ Exposure to NO_2_ during pregnancy can increase the lipid peroxidation level in the placenta, and cause abnormal growth and embryonic death.^[29]^ PM_10_ can directly enter the airway causing birth defects, and increase DNA adductions through the circulatory system.^[27]^ However, some studies have revealed no significant association between the air pollutants SO_2,_ NO_2,_ and PM_10_ and birth defects .^[5,7,8,30,31]^

The advantages of this study are as follows: First, this study demonstrated the association between air pollution and birth defects at the provincial level in China by selecting four geographical locations in Hunan province. To date, there is no similar research at the provincial level in China. Second, some studies have reported that exposure during the first trimester was associated with an increased risk of birth defects.^[9,13,32]^ Other studies suggested that third trimester exposure resulted in greater effects.^[11]^ It is still unknown whether the period of peak effect differs during the pregnancy stages. Thus, we determined the relationship between the three pregnancy stages and birth defects. Furthermore, we subdivided pregnancy into 3 month sections. In our study, a positive association was found between birth defects and SO_2_ exposure in the prophase of pregnancy, the first trimester and the third month before delivery. A positive association was also found between birth defects and NO_2_ exposure in the second month before pregnancy, the second and third month of pregnancy, and the third month before delivery. A positive association was also found between birth defects and PM_10_ exposure in the first month of pregnancy and in the third month before delivery.

Previous studies on this topic divided the exposure variables into categorical variables,^[20,21,30]^ while other studies used continuous exposure variables.^[6,7,23,31,33]^ In this study, we estimated the effect of air pollution as categorized exposure in quartiles, as categorical variables increased the statistical power.

In this study, the areas, years, season of conception, maternal age, gender of infant, total previous live births, and number of pregnancies were confounding factors. The season of conception and maternal age at conception were the most frequent confounders considered in this study. Social factors such as smoking, parental occupation, socioeconomic status, and educational level were not considered in this study, as this information was not available in the control group. Furthermore, the influence of social factors may be associated with maternal age.^[34]^ Gender of infant, total previous live births and number of pregnancies as the confounders in this study were also considered in other studies^[5,20,33]^ This study had some limitations. First, we did not use a distance-weighted calculation in the assessment of exposure, as the distance between maternal residence and the nearest monitoring station may produce inaccurate exposure estimates and thus lead to exposure misclassification. Improved exposure assessment methods, in particular more accurate spatial measurements or modeling are highly recommended for future birth defect research in relation to the effect of air pollution. Second, the exposure estimation was based on residential address, which may not adequately characterize the individual's actual exposure level. Furthermore, the recorded residence was not the only address during the pregnancy period. Reported rates of mobility between the time of conception and delivery vary widely, from approximately 9% to 32%.^[35]^ Third, this study was limited to reveal the impact of air pollution on birth defects, because other covariates, such as maternal disease history, coexisting comorbidities (eg, gestational hypertension, gestational diabetes, and cardiovascular disease), and prenatal care, may also influence the results.

## 5 Conclusions

Overall, our results indicated an association between exposure to air pollution and birth defects in Hunan province, China. The pollutants SO_2,_ NO_2,_ and PM_10_ had an effect on birth defects in the early first trimester. SO_2_ had the greatest effect in the prophase of pregnancy, while NO_2_ and PM_10_ had a greater effect in the late third trimester. We hope that health workers highlighted the impact of air pollution on birth defects when educating pregnant women. What is more, we hope the government focused on the impact of air pollution on birth defects now.

## Acknowledgments

The authors gratefully acknowledge all the members involved in the data collection of birth defect information, air quality and meteorological data in the four cities of Changsha, Changde, Yongzhou, and Huaihua.

## Author contributions

**Conceptualization:** Lili Xiong, Zenghui Xu.

**Data analysis:** Lili Xiong, Zenghui Xu.

**Data collection:** Lili Xiong, Zenghui Xu, Zhiyu Liu, Donghua Xie, Aihua Wang, Fanjuan Kong.

**Data curation:** Lili Xiong, Zenghui Xu, Donghua Xie, Aihua Wang, Fanjuan Kong.

**Formal analysis:** Lili Xiong.

**Funding acquisition:** Lili Xiong.

**Methodology:** Lili Xiong, Zenghui Xu.

**Project administration:** Lili Xiong, Hua Wang, Zhiyu Liu.

**Supervision:** Lili Xiong, Zenghui Xu, Zhiyu Liu, Hua Wang.

**Writing – original draft:** Lili Xiong, Zenghui Xu.

**Writing – review & editing:** Lili Xiong, Zenghui Xu, Hua Wang, Zhiyu Liu.
